# The influence of emotional intelligence on learning burnout in Chinese art college students: the chain mediation effect of self-acceptance and perceived stress

**DOI:** 10.3389/fpsyg.2024.1432796

**Published:** 2025-01-07

**Authors:** Meng Zhang, Lay Yoon Fah

**Affiliations:** Faculty of Social Sciences and Liberal Arts, UCSI University, Kuala Lumpur, Malaysia

**Keywords:** Chinese art students, emotional intelligence, learning burnout, perceived stress, self-acceptance

## Abstract

**Introduction:**

Art college students are under special pressure from a few sources, including study, employment, friends, emotions, family relations and other aspects. This can lead to a reasonable degree of learning burnout among art college students, which will have a negative impact on their physical and mental health, as well as their study and employment. However, there is a paucity of empirical studies on learning burnout among art students. Furthermore, the factors and mechanisms that contribute to learning burnout among art students remain unclear. The purpose of this study was to examine the relationship between emotional intelligence and learning burnout in Chinese art college students, and to identify the role of self-acceptance and perceived stress as sequential mediators.

**Methods:**

This study employed a completely random survey of three art universities in China to investigate the status of emotional intelligence, self-acceptance, perceived stress and learning burnout among art students and their relationships. The data was analyzed using an independent sample t-test and structural equation modelling.

**Results:**

A total of 678 valid samples were obtained from a study of Chinese art students. No significant differences were observed in the scores of emotional intelligences, self-acceptance, perceived stress, and learning burnout among participants of different genders. The results of the structural equation modelling indicated that emotional intelligence was negatively associated with learning burnout (*β* = −0.282, *p* < 0.001). The results indicated that self-acceptance (*β* = −0.140, *p* < 0.001) and perceived stress (β = −0.072, p < 0.001) had independent mediating effects. Furthermore, self-acceptance and perceived stress exhibited sequence mediating effects (*β* = −0.039, *p* < 0.001).

**Conclusion:**

The results of the study confirm that emotional intelligence is a significant factor in the learning burnout of art students. Furthermore, the findings demonstrate the mediating effect of self-acceptance and perceived pressure on this relationship. The findings provide a novel explanation for the mechanism underlying college students’ learning burnout.

## 1 Introduction

The sluggish global economic growth of recent years has also had a significant impact on China’s economy, which has traditionally been regarded as the world’s factory. This has resulted in a rise in the unemployment rate, intensified competition for employment among Chinese college students, and unprecedented pressure on these students to secure employment (Dong, 2023; Peng et al., 2024). Those with the same educational background are experiencing greater difficulty in finding satisfactory employment than previously (Dirks, 2023), and the prevalence of learning burnout among college students is on the rise (Chen et al., 2023). Compared to STEM students, art students in China face greater employment pressure. Art students tend to invest more in their education, have less clearly defined learning objectives, and are more susceptible to learning burnout.

In the field of learning psychology, learning burnout is defined as a negative emotional state resulting from a lack of interest or motivation in the learning process. This can manifest as feelings of boredom and exhaustion, which in turn lead to avoidance of learning activities (Ling et al., 2014). As with job burnout, learning burnout is a global phenomenon affecting students of diverse ages and academic disciplines (Liu et al., 2023). Although the issue has a significant negative impact on the mental health and academic achievement of college students, the issue of learning burnout has not been fully researched.

Additionally, Chinese art students represent a unique cohort within the realm of college students. Elkins (2001) argues that “Art cannot be taught or even nourished”. In fact, compared with most Science, technology, engineering, and mathematics (STEM) students, art students usually do not have certain skills that can be used immediately upon graduation, and thus have fewer job opportunities and greater employment pressure. They are also more likely to experience burnout while studying (Bernhard, 2021). In the context of the college entrance examination, the scores of art students within the same university are often comparatively lower, and the learning outcomes of art students in college are often more challenging to quantify. Furthermore, the employment expectations of art students are often less certain in comparison to practical majors such as medicine. Furthermore, art students may also suffer from learning burnout (Mei and Wei, 2022), yet no scholars have specifically studied the situation of learning burnout among art students. Consequently, it is an urgent task for the academic and theoretical circles to study the learning burnout of art students.

In their research on the factors influencing learning burnout, researchers will investigate and study the external factors and internal factors of individuals (Lee et al., 2020; Lubbadeh, 2020; Nápoles, 2022). At present, researchers have explored various external factors affecting learning burnout. Some scholars have investigated the influence of social support on learning burnout (Barratt and Duran, 2021; Velando-Soriano et al., 2020; Ye et al., 2021). Chen et al. (2023) explored the influence of parents’ educational expectations on the same phenomenon. Finally, Wu et al. (2022) examined the effect of parents’ educational expectations on learning burnout. A study by Rubino et al. (2009) examines the impact of stressors on learning burnout. A study by Yang et al. (2024) examines the influence of mobile phone addiction on learning burnout. In terms of exploring the internal factors that affect learning burnout, scholars mainly study the influence of internal motivation and personality on learning burnout (Amelia, 2022).

Only a few studies have looked at stress and burnout among art students. The empirical study of Liu et al. (2024) confirmed that art students’ intolerance to uncertainty and poor sleep quality have an impact on exam anxiety, indicating that art students’ mentality and emotional management ability are correlated with perceived learning pressure. Taking art students as the research object, Zhao (2023) confirmed that psychological capital plays an intermediary role in the influence of career expectation on employment anxiety, and the psychological capital of college students shows a significant positive correlation with self-acceptance (Yao et al., 2023). Meanwhile, students’ employment anxiety is closely related to perceived stress and burnout (Wright et al., 2023). Komarenko et al. (2024) proposed emotion-focused strategies for relieving stress in music majors, such as arousal reassessment and self-talk, which helped regulate mood and anxiety in music majors. Research by Lee et al. (2024) found that students who studied the arts reported higher levels of mental distress, stress, and time spent on academic work than their non-arts peers. This shows that art students face greater learning pressure and are more likely to have learning burnout.

However, in the field of job burnout, it has been demonstrated that individual emotional intelligence plays an important role in the development of job burnout (Cao et al., 2022; Han et al., 2022). Furthermore, emotional intelligence has been found to have a significant impact on teachers’ job burnout (Cece et al., 2022; Lucas-Mangas et al., 2022; Wang and Wang, 2022). Some studies have also indicated that the emotional intelligence of medical students may influence their learning burnout (Blanchard et al., 2021; Shariatpanahi et al., 2022). Most studies have explored how emotional intelligence of professionals or teachers directly affects burnout, but few have delved into the relationship between emotional intelligence and burnout of art students. This study aims to establish a model of the influence of emotional intelligence on learning burnout of Chinese art students.

### 1.1 The role of self-acceptance as a mediator between emotional intelligence and learning burnout

Previous studies have demonstrated that an individual’s emotional intelligence level is positively correlated with self-acceptance. Lu et al. (2022) conducted a study with psychiatric nurses in Shandong, confirming that emotional intelligence can influence self-acceptance. The theoretical work of Deligkaris et al. (2014) highlighted a direct relationship between individual cognition and the onset of job burnout. An empirical study by Alfuqaha et al. (2019) also demonstrated a significant association between core self-evaluation and burnout. Additionally, self-acceptance based on self-cognitive evaluation has been demonstrated to influence burnout (Dirks, 2023). Furthermore, self-acceptance is not only directly negatively correlated with burnout, but also functions as an intermediary variable between perfectionism and burnout (Hill et al., 2008). The analysis of job burnout as a source of learning burnout has revealed that emotional intelligence and self-acceptance are important individual internal factors of job burnout. Consequently, it can be hypothesized that the emotional intelligence and self-acceptance of Chinese art students will also affect learning burnout. The capacity to regulate one’s emotions, or emotional intelligence, can not only facilitate an individual’s self-understanding and evaluation, but also emotionally promote an individual’s positive acceptance of their actual self (Lu et al., 2022). In other words, emotional intelligence can promote individual self-acceptance. When considered in the context of the above theoretical analysis, which suggests that emotional intelligence and self-acceptance may affect learning burnout, it becomes apparent that academic exhaustion can be significantly influenced by emotional quotient, with self-validation serving as an intermediary element in the correlation between emotional quotient and academic exhaustion.

### 1.2 The role of perceived stress as a mediator between emotional intelligence and learning burnout

Art students must learn a wide range of content, relatively unclear learning objectives, various art skills, and relatively unclear employment prospects. Additionally, the learning environment is subject to high levels of pressure, which has been identified as a significant contributing factor to learning burnout (Lee et al., 2024; Gong et al., 2023). Zhou et al. (2024) demonstrated that stress has a significant impact on the learning burnout of on-the-job postgraduates. Furthermore, Melguizo-Ibáñez et al. (2023) found that stress also had a significant impact on learning burnout among college students majoring in physical education. The findings of Lea et al. (2019) indicate that emotional intelligence can assist individuals in effectively managing the adverse effects of stress. Furthermore, groups with high emotional intelligence are less likely to perceive pressure. In a study published in 2019, Choi et al. (2019) demonstrated that job stress acts as a mediator between emotional intelligence and job burnout. In a subsequent study published in 2021, Yusoff et al. (2021) confirmed that study stress also acts as a mediator between emotional intelligence and learning burnout among medical students. In line with the definition theory of causality (Pearce and Vandenbroucke, 2016; Pearl et al., 1995), the present study postulated that the learning stress experienced by art college students functions as an intermediary factor. This investigation delved into the effect of emotional intelligence on academic burnout, focusing specifically on the emotional intelligence levels of art college students. The examination of emotional intelligence’s influence on academic burnout among Chinese art students involved an assessment of the mediating roles played by self-acceptance and perceived stress.

### 1.3 The chain mediating role of self-acceptance and perceived stress between emotional intelligence and learning burnout

Self-acceptance is not only an important predictor of learning burnout, but also closely related to an individual’s perceived stress (Sun et al., 2019; Xu et al., 2017). The study of Rodriguez et al. (2015) confirmed that individuals with a high degree of self-acceptance would have lower perceived pressure. Self-acceptance is associated with a reduction in self-demanding and excessive expectations (Carson and Langer, 2006). When individuals can accept their shortcomings and inadequacies, they are less likely to experience stress and anxiety due to their imperfections (Cohen, 2019). This can facilitate their ability to face challenges in life, thereby reducing the perceived stress. Secondly, self-acceptance can enhance self-esteem and self-confidence (Rosida and Taqiyah, 2024). When individuals can genuinely accept themselves and recognize their uniqueness and worth, their self-esteem and self-confidence levels increase. This confidence and self-esteem can assist in better coping with stress (Abdulghani et al., 2020; Li and Lyu, 2021).

When confronted with pressure, maintaining an attitude of self-acceptance enables a more objective analysis of the problem and the identification of solutions, rather than being overwhelmed by the pressure (Cho et al., 2014). Based on these findings, this study hypothesis that self-acceptance among art college students may negatively predict perceived stress. Considering the hypotheses, we propose the notion that the concepts of self-acceptance and perceived pressure serve as mediators in the complex dynamics of the relationship between emotional intelligence and the experience of learning burnout, indicating a more profound and nuanced comprehension of the mechanisms involved in this correlation.

This mechanism has been corroborated by relevant studies. Sun et al. (2019) employed a sample of 307 Chinese special education teachers to substantiate the chain mediating role of self-acceptance and perceived stress in the relationship between mindfulness and job burnout. However, the research object of this research is special education teachers, and the factor affecting learning burnout is mindfulness. Currently, no scholars have studied the chain mechanism of self-acceptance and perceived stress of college students in the relationship between emotional intelligence and learning burnout. This study will seek to address this issue.

Since there are more women than men of art students in China, gender may influence the results of the study, so the role of gender in previous studies was reviewed. Fida et al. (2018) found that the EQ of female students was ahead of that of male students, but Ali et al. (2021) got the opposite result, that is, the EQ of male students was higher than that of female students. In terms of students’ self-acceptance level, Aykut Ceyhan and Ceyhan (2011) found no significant difference between males and females. Graves et al. (2021) found that women showed higher levels of stress than men. A study by Fiorilli et al. (2022) found that female students exhibited higher levels of burnout than male students. However, the study of Onuoha and Akintola (2016) believes that the impact of student gender on burnout is uncertain, that is, the burnout level of students of different genders is not necessarily different.

### 1.4 Conceptual framework and hypothesis

**Figure 1:**
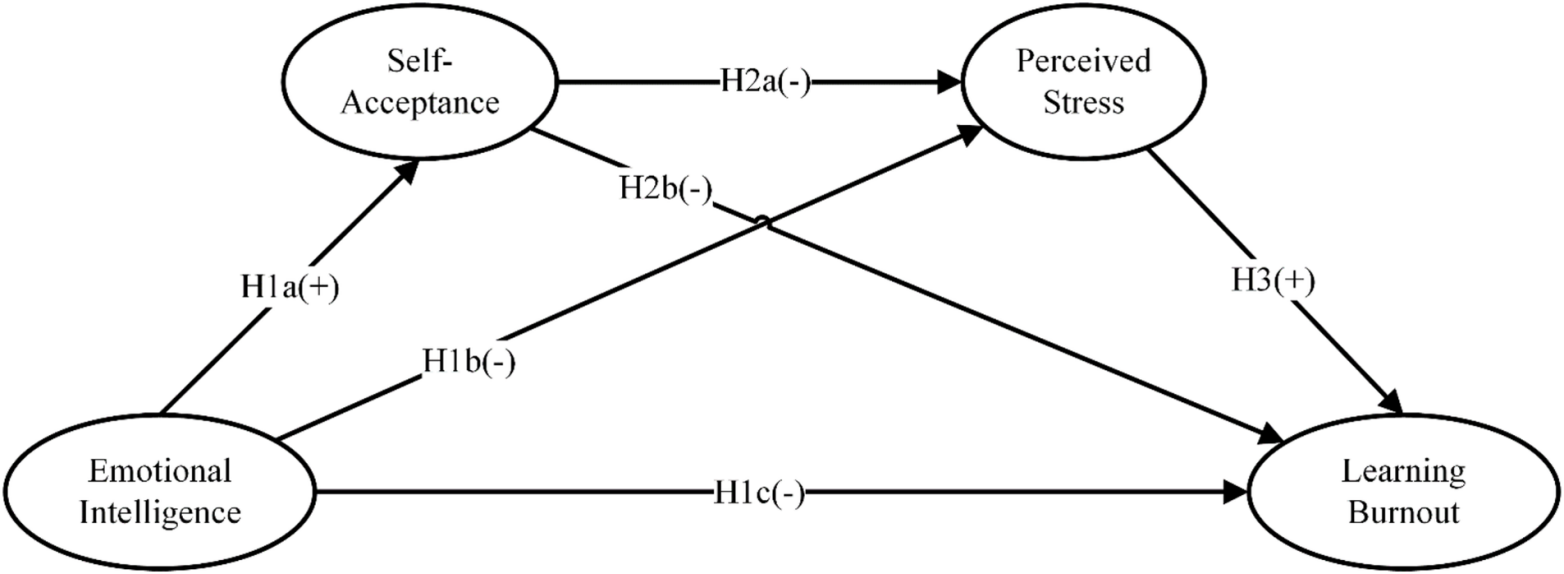
Conceptual framework.

In this model, the input variable is the emotional intelligence of college students. Self-acceptance and pressure are process variables in the influencing mechanism, and learning burnout is the risk factor. Based on the conceptual framework shown in Figure 1, this study proposes the following series of hypotheses: In this model, the internal emotional intelligence of college students is the input variable, self-acceptance and perceived pressure are the process variables in the influencing mechanism, and learning burnout is the risk factor. Based on the conceptual framework shown in Figure 1, this study proposes the following series of hypotheses:

*H1a*: The impact of emotional intelligence on self-acceptance among Chinese art college students is notably positive.

*H1b*: There exists a significant adverse relationship between the emotional intelligence of Chinese art students and their perceived stress.

*H1c*: The emotional intelligence of Chinese art students is found to have a substantial negative influence on learning burnout.

*H2a*: The self-acceptance levels of Chinese art students demonstrate a significant negative correlation with perceived stress.

*H2b*: The self-acceptance of Chinese art students is significantly associated with reduced learning burnout.

*H3*: The level of perceived stress experienced by Chinese art students is a positive predictor of learning burnout.

*H4*: The self-acceptance exhibited by Chinese art students plays a mediating role in the association between emotional intelligence and the experience of learning burnout.

*H5*: The perceived stress experienced by Chinese art students serves as a mediator in the correlation between emotional intelligence and the occurrence of learning burnout.

*H6*: The self-acceptance and perceived stress among Chinese art students are found to sequentially mediate the relationship between emotional intelligence and learning burnout.

## 2 Methodology

### 2.1 Participants

Three art universities in Beijing, which have cooperative relations with the university where the author works, were selected for a questionnaire survey. According to the ID number in the academic affairs system of the university, 250 students from the three universities were selected by completely random method, and the questionnaire link was sent to their student email, and 678 valid questionnaires were collected. The respondents were all art undergraduates, aged between 18 and 23. Of these, 246 were men and 432 were women. All participants involved in this research were required to review and authorize an informed consent document prior to completing the survey. Only respondents who chose “agree” were permitted to participate in the survey. Furthermore, G*power 3.1.9.7 software was employed to determine the requisite minimum sample size of 550 (f^2^ = 0.02, power = 0.80, *α* = 0.05), and the actual sample size obtained in this study exceeded 550.

### 2.2 Measures

In this study, four measuring instruments from different sources were used to measure the study variables. To reduce the bias caused by common measurement methods, Likert scales with different points were used as measuring tools. The initial sources of these four scales and their reliability and validity test results are as follows:

#### 2.2.1 Emotional intelligence

Emotional Intelligence was assessed utilizing a psychometrically robust measurement tool devised by Wong and Law (2002), encompassing four sub-dimensions comprising a total of 16 items, with four items allocated to each sub-dimension. Shi and Wang (2007)validated that the scale exhibited commendable reliability and validity among Chinese university students. The scale was employed in appraising the emotional intelligence of Chinese art students. An illustrative item from this assessment instrument is: “I possess a profound understanding of the reasons behind my feelings on most occasions.” These items underwent assessment through the employment of the Likert 7-point scale (1 = “very inconsistent,” 7 = “very consistent”), with elevated scores denoting heightened emotional intelligence within this domain. Confirmatory factor analysis was employed in this inquiry to conceptualize the sample data utilizing the predetermined factor structure of the scale, culminating in the acquisition of the fitting index as delineated below: The findings of the analysis reveal that the model aligns well with the data. The ensuing values were ascertained: *χ*^2^ = 544.930, df = 100, χ^2^/df = 5.449, IFI = 0.946, TLI = 0.935, CFI = 0.946, SRMR = 0.065, RMSEA = 0.081. The Cronbach’s Alpha coefficient of the entire scale was 0.926, indicating that the Emotional Intelligence measurement scale exhibited a satisfactory degree of reliability and validity.

#### 2.2.2 Self-acceptance

The self-acceptance subdimension of Ryff and Singer's (2010) Mental Health Scale (SPWB) was used to measure self-acceptance. The Chinese version of this scale, validated by Li (2014), has been shown to have good reliability and validity. The subdimension comprises five items that assess the individual’s acceptance of both positive and negative aspects of their self-perception. The self-acceptance subscale includes the following sample items: “When I compare myself to friends and acquaintances, it makes me feel good about who I am.” and “In general, I feel confident and positive about myself.” The five items include a reverse measurement problem, “Everyone has their weaknesses, but I seem to have more than my share,” which needs to be reversed coded after data collection. The self-acknowledgment subscale is evaluated on a five-point Likert scale, where 1 signifies intense dissent and 5 denotes strong concurrence. A higher score on the self-acceptance subscale indicates a higher level of acceptance of respondents’ positive or negative personality traits, while a lower score indicates a lack of satisfaction with oneself. The CFA model fitting index of the scale in this study is: *χ*^2^ = 9.684, df = 5, χ^2^/df = 1.937, IFI = 0.996, TLI = 0.992, CFI = 0.996, SRMR = 0.015, RMSEA = 0.037, Cronbach’s Alpha value is 0.833.

#### 2.2.3 Perceived stress

The Simplified Chinese rendition of the 10-item Perceived Stress Scale (SCPSS-10) is employed to assess perceived stress. Lu et al. (2017) examined the dependability and credibility of the scale in Chinese university students and discovered it to be efficacious. The SCPSS-10 comprises two facets, encompassing the facets of Perceived Helplessness (item1, item2, item3, item6, item9, item10); and sub-divisions of Perceived Self-Efficacy (item4, item5, item7, and item8). It is important to acknowledge that the four items relating to the Perceived Self-Efficacy sub-division are reverse scoring inquiries, necessitating reverse coding prior to data scrutiny. To illustrate a positive scoring item for SCPSS-10, refer to the subsequent inquiry: “Over the past month, how often have you experienced distress due to unforeseen events?” Conversely, for a negative scoring item, consider the subsequent question: “Over the past month, how often have you perceived that circumstances were in your favor?” In this investigation, the SCPSS-10 was rated using a 5-point Likert scale, with 0 denoting “never” and 4 denoting “frequently.” The fit indices of the confirmatory factor analysis (CFA) model for the scale in this research are as follows: *χ*^2^ = 191.399, df = 34, χ^2^/df = 5.629, IFI = 0.943, TLI = 0.925, CFI = 0.943, SRMR = 0.054, RMSEA = 0.083. The Cronbach’s Alpha stands at 0.850.

#### 2.2.4 Learning burnout

This paper employs the Learning Burnout Scale developed by Schaufeli et al. (2002), based on the Maslach Job Burnout Questionnaire (Maslach et al., 1997), to assess the learning burnout of college students participating in the study. The scale comprises 16 items and assesses learning burnout from three dimensions: exhaustion, learning cynicism, and reduced efficacy. The 16-item learning burnout scale, as developed by Zhang and Fah (2024), performed poorly in Chinese art college students. However, the 14-item 3-dimensional structure, which was created by removing two items, demonstrated good reliability and validity. In this study, the 14-item Learning Burnout Scale was used to measure learning burnout. Confirmatory factor analysis was used to model the sample data with the preset factor structure of the scale, and the following fitting index was obtained: The results of the confirmatory factor analysis indicate that the model fits the data well. The following indices were obtained: *χ*^2^ = 396.585, df = 74, *χ*^2^/df = 5.359, IFI = 0.953, TLI = 0.943, CFI = 0. The resulting reliability and validity indices were as follows: 0.953 for Cronbach’s Alpha, 0.064 for SRMR, 0.080 for RMSEA, and 0.889 for the alpha coefficient. These indices indicate that the scale for measuring learning burnout has satisfactory reliability and validity.

### 2.3 Procedure and data analysis

The questionnaire included four measuring instruments (46 items) and gender, and no other demographic variables were collected. Prior to distribution, the questionnaire was reviewed and evaluated by experts from various fields, including psychology, music, painting, and education. This process ensured the content was suitable for Chinese art students to understand and answer. The questionnaire was created using the Questionnaire Star platform, and the link was generated. Participants were selected from the student roster at three selected arts universities using a random selection process. The questionnaires were reviewed by administrators at the three universities, allowing data collection. The hyperlink to the questionnaire was distributed via the student email platform. Before responding to the questionnaire, students were mandated to endorse an informed consent document. Subsequently, upon fulfilling this requirement, they were granted permission to proceed with the questionnaire. The introductory declaration in the questionnaire explicitly indicated the absence of correct or incorrect responses, allowing students to complete it based on their personal sentiments.

The sample data of this study was completed from November to December 2023, and the data filled in by students was saved in the questionnaire star system. The average filling time of the questionnaire was 8 min.

SPSS26.0, AMOS26.0 and Smart PLS 4.0.8.7 software were used for data processing and analysis. Among them, SPSS26.0 was used for descriptive statistical analysis, reliability analysis, common method bias test, calculation of average scores of each measuring instrument, and independent sample T-test for scores of different gender groups. Confirmatory factor analysis was conducted utilizing AMOS26.0 to validate the structural integrity of each measuring device. Smart PLS 4.0.8.7 was used to test the common variance bias, test the hypotheses in the conceptual model, and analyze the mediating effects.

## 3 Results

### 3.1 Common method bias test

The study was designed to control for common methodological bias procedurally, according to the suggestions of Rodríguez-Ardura et al. (2020). For instance, Likert scales with different rating levels were used in question preparation and respondents were informed that their responses would be kept anonymous. Furthermore, respondents were advised that the questionnaire was not intended to be an assessment or test, but instead, a tool for gathering data according to their individual circumstances. However, the scales measuring the four major variables in this study were all presented to respondents in the same questionnaire, and these data were self-reported by respondents. This may still result in common methodological bias (Podsakoff and Organ, 1986). To circumvent the impact of regular method bias on the research findings, this investigation utilizes statistical approaches to examine common method bias. Primarily, the singular aspect examination devised by Harman is employed to assess common method bias. The quantity of elements with an eigenvalue surpassing 1 is 10. The percentage of variance attributed to the initial factor is 30.646%, which falls below 50%. This suggests that common method bias is not a noteworthy concern (Jordan and Troth, 2020; Philip and Dennis, 1986). Subsequently, the Full Collinearity Assessment Approach proposed by Kock and Lynn (2012) is employed to assess the common variance deviation. The variance inflation factor (VIF) obtained is 1.698, 1.462, 1.554 and 1.539, respectively. All these values are below the critical value of 3.3 suggested by Kock (2015), the results suggest that there is no notable issue of common method bias present in the sample data used in this research.

### 3.2 Analysis of differences between groups

The independent sample t-test was utilized to evaluate the differences between groups of varying genders. The findings are displayed in Table 1, demonstrating the absence of noticeable variations between male and female cohorts in emotional intelligence, self-acceptance, perceived stress, and learning burnout. From the effect size level, the effect size of the difference between the sexes was less than 0.01, which did not reach the level of a small effect (*η*^2^ > 0.01).

**Table 1:** Analysis of differences between gender.

Variables	Gender	*N*	Mean	SD	*t*	*P*	*η*^2^
Emotional intelligence	Male	246	5.05	0.76	1.890	0.059	0.005
	Female	432	4.93	0.85			
Self-acceptance	Male	246	3.60	0.53	−1.066	0.287	0.002
	Female	432	3.65	0.52			
Perceived stress	Male	246	1.40	0.57	1.689	0.092	0.004
	Female	432	1.32	0.55			
Learning burnout	Male	246	3.50	0.81	0.941	0.347	0.001
	Female	432	3.44	0.81			

*η*^2^ = 0.01 means small effects, *η*^2^ = 0.06 means medium effects, and *η*^2^ = 0.14 means large effects (Lakens, 2013).

### 3.3 Structural model assessment

The first step was to run a collinearity test using the PLS algorithm on the conceptual model presented in this paper. This was done using the PLS software, version 4.0.8.7. The findings of this examination are illustrated in Table 2. When Perceived Tension is regarded as the outcome variable, the Variance Inflation Factor of both predictor variables is 1.465. When Educational Exhaustion is a predictor variable, the VIF coefficients of the three independent variables are 1.648, 1.634, and 1.552, respectively, all falling below the crucial threshold of 5 recommended by Warne (2017), suggesting the absence of a noteworthy multicollinearity concern.

**Table 2:** Collinearity statistics.

Independent variable	Dependent variable	
	Perceived stress	Learning burnout
Emotional intelligence	1.465	1.648
Self-acceptance	1.465	1.634
Perceived stress		1.552

**Figure 2:**
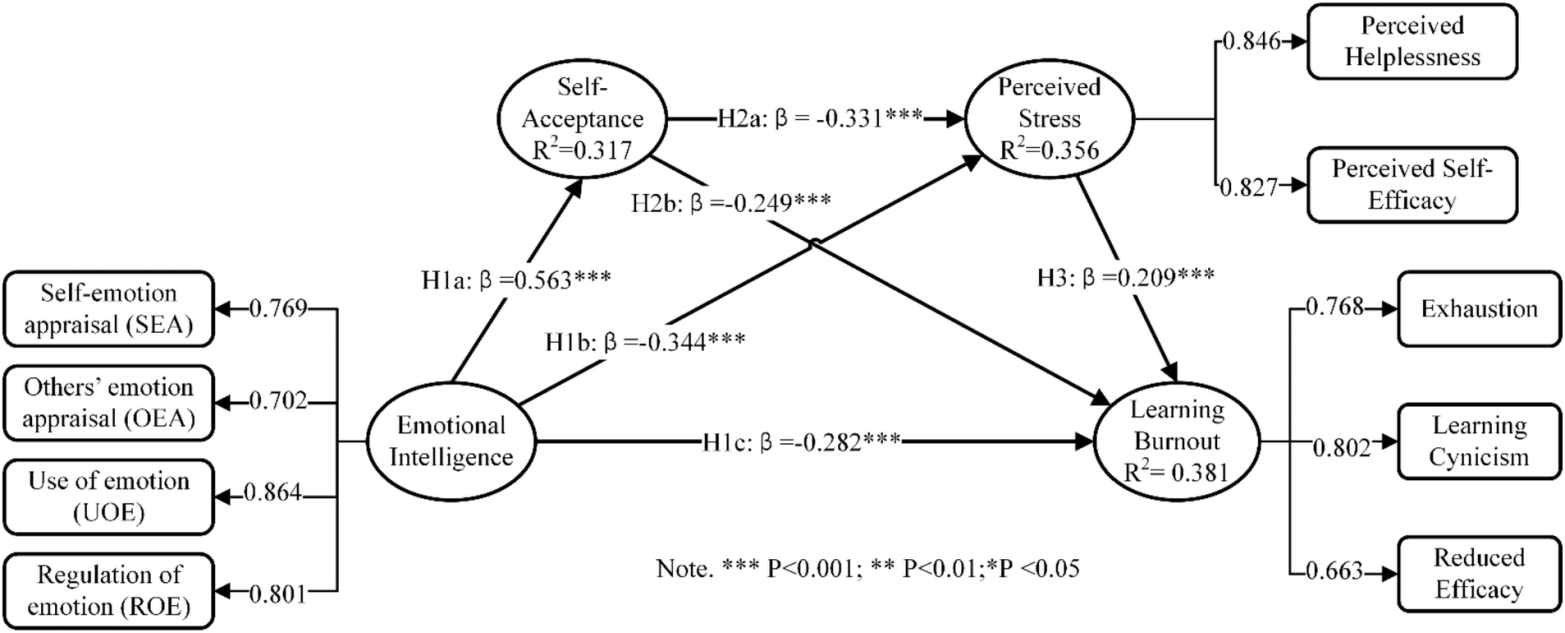
Test results of the structural model.

The Bootstrap method was then employed to assess the significance of each path coefficient in the structural model. The bootstrap samples were set to 5,000 and 95% confidence intervals were selected with bias correction. The estimated results of the path coefficient are presented in Figure 2 and Table 3.

**Table 3:** Hypothesis test results.

Hypothesis	Path	*β*	SE	*T*	*P*	*f*^2^	95%CI	Supported
H1a	EI → SA	0.563	0.03	20.170	<0.001	0.465	[0.501, 0.612]	yes
H1b	EI → PS	−0.344	0.03	10.040	<0.001	0.125	[−0.406, −0.271]	yes
H1c	EI → LB	−0.282	0.04	7.658	<0.001	0.078	[−0.349, −0.205]	yes
H2a	SA → PS	−0.331	0.03	9.800	<0.001	0.116	[−0.397, −0.265]	yes
H2b	SA → LB	−0.249	0.04	6.498	<0.001	0.061	[−0.326, −0.177]	yes
H3	PS → LB	0.209	0.04	5.420	<0.001	0.045	[0.128, 0.281]	yes

EI, emotional intelligence; SA, self-acceptance; PS, perceived stress; LB, learning burnout. *f*^2^ = 0.02 means small effect, *f*^2^ = 0.15 means medium effects, and *f*^2^ = 0.35 means large effects (Myors and Murphy, 2023).

According to Hypothesis H1a, there is a positive relationship between emotional intelligence and self-approval, supported by a path coefficient of 0.563 and a 95% confidence interval of [0.501, 0.612]. The *p*-value from the consequence examination is less than 0.001, which backs up the proposition. Proposition H1b declares that emotional quotient has a noteworthy adverse effect on perceived tension, with a path coefficient of −0.344 and a 95% assurance interval of [−0.406, −0.271]. The *p*-value of its consequence examination is less than 0.001, indicating that Proposition H1b is backed up. Proposition H1c postulates that emotional quotient has a significant adverse effect on learning fatigue, with a path coefficient of −0.282 and a 95% assurance interval of [−0.349, −0.205]. The p-value of its consequence examination is less than 0.001, indicating that Proposition H1c is supported. Proposition H2a is that self-approval has a significant adverse impact on perceived pressure, with a path coefficient of −0.331 and a 95% assurance interval of [−0.397, −0.265]. The p-value of the consequence examination is less than 0.001, indicating that H2a is supported. Proposition H2b is that self-approval has a significant adverse effect on learning fatigue, with a path coefficient of −0.249 and a 95% assurance interval of [−0.326, −0.177]. The *p*-value of the consequence examination is less than 0.001, indicating that H2b is supported. Proposition H3 is that perceived tension has a significant positive impact on learning fatigue, with a path coefficient of 0.209 and a 95% assurance interval of [0.128, 0.281]. The p-value of the consequence examination is less than 0.001, indicating that Proposition H3 is supported.

In line with the guidance set out by Hair et al. (2021), the predictive power of a model can be described as strong, medium, or weak depending on whether the *R*^2^ value of the endogenous latent variable reaches 0.75, 0.50 or 0.25. As illustrated in Figure 2, the *R*^2^ values for the three endogenous variables, namely Self-Acceptance, Perceived Stress and Learning Burnout, are 0.317, 0.356 and 0.381, respectively. The values lie within the range of 0.25 to 0.50, suggesting that the model’s explanatory capacity is moderately limited.

### 3.4 Analysis of mediating effects

The utilization of the Bootstrap method, as integrated within Smart-PLS 4.0.8.7 software, is necessary for evaluating the importance of the mediation path coefficient. It is recommended to configure the bootstrap samples to 5,000 and opt for a 95% bias-corrected confidence interval. The results are shown in Table 4.

**Table 4:** The results of the mediation effect test.

Path	*β*	SE	*t*	*P*	95% CI	VAF
**Specific indirect effect**						
EI → SA → LB	−0.140	0.02	5.919	<0.001	[−0.190, −0.097]	26.3%
EI → PS → LB	−0.072	0.02	4.606	<0.001	[−0.103, −0.042]	13.5%
EI → SA → PS → LB	−0.039	0.01	4.518	<0.001	[−0.056, −0.023]	7.3%
**Total indirect effect**						
EI → LB	−0.251	0.03	9.927	<0.001	[−0.301, −0.200]	47%
**Direct effect**						
EI → LB	−0.282	0.04	7.658	<0.001	[−0.39, −0.205]	53%
**Total effect**						
EI → LB	−0.533	0.03	20.187	<0.001	[−0.577, −0.472]	

EI, emotional intelligence; SA, self-acceptance; PS, perceived stress; LB, learning burnout.

The coefficient representing the indirect impact of emotional intelligence on learning burnout through self-acceptance was −0.140, accompanied by a 95% confidence interval of [−0.190, −0.097], and a p-value below 0.001 indicating statistical significance. This indirect effect value explained 26.3% of the overall impact emotional intelligence has on learning burnout. Similarly, the indirect effect value attributed to emotional intelligence on learning burnout through perceived stress is −0.072, supported by a 95% confidence interval of [−0.103, −0.042] and a *p*-value below 0.001. This specific indirect effect size contributes to 13.5% of the total impact of emotional intelligence on learning burnout. Furthermore, the indirect effect value denoting the influence of Emotional Intelligence on Learning Burnout through Self-Acceptance and Perceived Stress is −0.039, with a 95% confidence interval of [−0.056, −0.023]. The *p*-value derived from the significance test was below 0.001, highlighting that this indirect effect value explains 7.3% of the total impact of Emotional Intelligence on Learning Burnout.

## 4 Discussion

This research introduces a conjecture drawn from existing literature suggesting that self-acceptance and perceived stress serve as mediators in the impact of emotional intelligence on learning burnout. The findings of this study substantiate the idea that self-acceptance and perceived stress function as mediators, thus confirming the notion that self-acceptance and perceived stress operate sequentially in this process.

The results of the study suggest that there is no notable disparity in the level of learning exhaustion experienced by individuals of varying genders. Furthermore, art students present a moderate degree of learning burnout. In the context of an increasingly competitive society, schools and families have increasingly high expectations of students, which can lead to learning burnout occurring from primary school students to research students (Chen et al., 2023; Tang et al., 2021). As evidenced by Teuber et al. (2021) and Wen et al. (2023), the issue of learning burnout among art students is also a significant concern (Gilbert, 2021; Payne et al., 2020). Some research studies have suggested that there is no discrepancy between genders when it comes to experiencing learning burnout, as evidenced by findings from studies conducted by Palupi and Findyartini (2019), Shin et al. (2022), and Vinter et al. (2021). Conversely, Herrmann et al. (2019) have demonstrated that there are differences between the sexes. The findings of this study indicate that there is no discernible gender difference in the learning burnout of art college students, which is consistent with the results of Shin et al. (2022). The discrepancy in the conclusions regarding gender differences in learning burnout may be attributed to the differing research objects and research tools employed in the respective studies.

### 4.1 The mediating effect of self-acceptance

The findings demonstrate that emotional intelligence exerts a notable beneficial impact on self-acceptance (*β* = 0.563), displaying a substantial effect size (*f*^2^ = 0.456). This implies that emotional intelligence significantly enhances self-acceptance. This aligns with Lu et al.'s (2022) assertion that emotional intelligence plays a crucial role in fostering self-acceptance among psychiatric nurses. Emotional intelligence is described as the ability to perceive, comprehend, and regulate one’s own emotions as well as those of others (MacCann et al., 2020). When individuals can accurately identify their emotions and understand the reasons behind them, they are more likely to see themselves in a positive, rational way (Wang et al., 2020). This auto-awareness facilitates a reduction in unfavorable self-evaluations, thereby enhancing self-acceptance (Sun et al., 2019). The results of the descriptive statistics indicate that the overall emotional intelligence of art students exhibits a trend of medium to high, with a corresponding medium to high score for self-acceptance, exhibiting consistency. Individuals with high emotional intelligence tend to exhibit superior emotional regulation (Megías-Robles et al., 2019). They are adept at effectively coping with negative emotions such as stress, anxiety, and frustration, and maintaining emotional stability and balance (Zhoc et al., 2020). This capacity to manage emotions serves to reduce self-doubt and feelings of inferiority, thereby facilitating the acceptance of one’s imperfections and flaws (Lu et al., 2022). Those with high emotional intelligence are generally better at socialising and establishing good interpersonal relationships with others (Zhoc et al., 2020). This helps individuals gain support and recognition in social environments, thus enhancing their sense of self-worth and self-acceptance (Shuo et al., 2022). Those with high emotional intelligence tend to exhibit higher self-esteem and confidence (Pérez-Fuentes et al., 2019). They demonstrate a clear understanding of their abilities and values and are less susceptible to negative external evaluation (Sun et al., 2021). This self-esteem and confidence serve as an important foundation for self-acceptance, enabling individuals to view their strengths and weaknesses in a more positive light. The findings revealed that self-acceptance was found to have a notable adverse predictive impact on learning burnout (*β* = −0.249), displaying a minor effect size (*f*^2^ = 0.061). This indicates that self-acceptance could potentially aid in reducing learning burnout to some degree. Individuals who possess a strong sense of self-acceptance might encounter a lower level of learning burnout. These results align with the research findings of Alfuqaha et al. (2019). Those who can acknowledge and accept their strengths and weaknesses are more likely to maintain a balanced and positive state of mind (Waxman et al., 2022). This positive attitude helps individuals to avoid burnout when faced with learning challenges and difficulties (Liu, 2023). Self-acceptance helps individuals form positive self-perception and value evaluation (Ueno et al., 2019). Such self-esteem and confidence can stimulate individuals’ enthusiasm and motivation for learning, make them more engaged in learning, and reduce the possibility of learning burnout (Chen et al., 2023). The outcomes of the mediation analysis performed utilizing the bootstrap technique reveal that the mediating path coefficient relating emotional intelligence to learning burnout is −0.140, showing statistical significance. This implies that self-acceptance functions as an intermediary variable in the association between emotional intelligence and learning burnout.

### 4.2 The mediating effect of perceived stress

Empirical evidence suggests that emotional intelligence has a notable impact on perceived stress. The results of this specific study unveiled that emotional intelligence exerts a significant negative impact on the perception of stress, as validated by the statistically significant beta coefficient of −0.344, indicating a moderate-to-small effect size measured by the *f*^2^ value of 0.125. This implies that emotional intelligence may play a role in moderating perceived stress levels. This finding aligns with the conclusions of previous research by Lea et al. (2019). Those with high emotional intelligence can manage their emotions effectively, and when they encounter difficulties in their studies, they are able to remain calm and not succumb to excessive anxiety or pressure (Fiorilli et al., 2020). Individuals who possess elevated levels of emotional intelligence demonstrate an enhanced capacity to effectively navigate decision-making processes (Alzoubi and Aziz, 2021). In the context of academic pressure, they can make well-informed decisions and adopt effective coping strategies, thereby reducing the negative impact of academic pressure on them. The results indicated that perceived stress had a significant positive predictive effect on learning burnout (β = 0.209), with a small effect size (*f*^2^ = 0.045). This suggests that perceived stress is a risk predictor of learning burnout, with the stronger the perceived stress, the more likely it is to produce learning burnout. Excessive learning pressure has been demonstrated to result in psychological burden and tension (Zieliński et al., 2021). This can lead to feelings of fatigue and a lack of ability to cope with learning tasks, which in turn can result in a loss of interest and motivation in learning (Yang et al., 2022). It is also evident that stress associated with study can give rise to several physical health issues, including a lack of sleep and an irregular diet (Alotaibi et al., 2020). These physiological problems serve to impede the ability of students to concentrate on their studies, thereby increasing the likelihood of learning burnout (Allen et al., 2021; Brubaker and Beverly, 2020). In high-stress environments, students may prioritize test-taking skills and short-term performance improvement (Mappadang et al., 2022). This utilitarian approach to learning may result in students feeling disengaged from the learning process. The outcomes of the mediation analysis carried out utilizing the bootstrap technique demonstrate that the mediating path coefficient in the context of the association between emotional intelligence and learning burnout is −0.072, a statistically meaningful finding. This implies that the perceived stress serves as a mediating factor linking emotional intelligence with learning burnout.

### 4.3 The chain mediating effects

The findings suggested that self-acceptance exhibited a notable adverse predictive impact on the perception of stress, as indicated by the beta coefficient of −0.331, with a moderate to small effect size (*f*^2^ = 0.116). This suggests that self-acceptance may be an effective approach to reducing an individual’s perceived stress. The findings suggest that there is a sequential relationship where self-acceptance and perceived stress act as intermediaries between emotional intelligence and learning burnout, indicating a complex interplay among these variables in the context of academic settings. This is in line with previous findings that emotional intelligence significantly positively predicts self-acceptance, and that perceived stress significantly positively predicts learning burnout. The mediation effect analysis of the Bootstrap method also corroborated this conclusion, with a coefficient of chain mediation of −0.039, which was statistically significant. This suggests that self-acceptance and perceived stress are intermediary factors in the relationship between emotional intelligence and learning burnout, thus validating the findings of Sun et al. (2019).

### 4.4 Research significance

The paper presents a theoretical exploration of emotional intelligence as a potential influencing factor in college students’ learning burnout. The study also explores the intermediary function of self-acceptance and perceived pressure in the correlation between emotional intelligence and learning burnout. This makes a theoretical contribution to the study of learning burnout of college students, with a particular focus on Chinese art undergraduates. Furthermore, at present, there are no scholars specializing in the study of learning burnout of Chinese art students. This study therefore represents a significant contribution to this field. In practice, Chinese university management institutions can intervene with college students’ learning burnout according to the conclusions of this study. The results of the research suggest that fostering emotional intelligence in university students shows potential in enhancing self-acceptance, decreasing perceived stress, and ultimately alleviating academic burnout.

### 4.5 Limitations and future directions

Due to the constraints of time, funding and resources, this study is subject to certain limitations. Primarily, all the data presented in this study were self-reported by participants, and no additional pertinent data was available for verification. The reliability of self-reported data is often influenced by social expectations. In the future, data collection could be enhanced by independent assessment, mutual assessment, or the use of electronic instruments. For instance, the stress-related data presented in this study could be measured by means of psychometric instruments. Secondly, the current investigation utilized a cross-sectional design, a methodological choice that limits the capacity to examine the associations among emotional intelligence, self-acceptance, perceived stress, and learning burnout through a longitudinal experimental methodology. The assumption of causality was posited drawing on existing scholarly works, with this study solely focusing on the evaluation of the theoretical framework, without analyzing other potential models. In the future, the design of longitudinal experiments involving time can be considered to verify causality by measuring changes in these variables over different times.

## 5 Conclusion

The research postulates that there is no substantial difference in the level of learning burnout encountered by art students across diverse genders. It has been observed that the emotional intelligence of college students plays a crucial role not only in directly influencing learning burnout but also in indirectly impacting it through factors such as self-acceptance and perceived pressure. Moreover, it has been noted that self-acceptance and perceived pressure exhibit distinct mediating effects as well as chain mediating effects on the phenomenon of learning burnout. Additionally, it has been found that as the emotional intelligence of college students increases, so does their level of self-acceptance, resulting in decreased perceived pressure and subsequently leading to a lower degree of learning burnout. Based on these findings, art colleges and universities should pay more attention to the cultivation of students’ emotional intelligence in the future education practice. It is also necessary to let art students better understand their major through necessary courses, to accept their identity as art students. In addition, more business cooperation should be sought to allow art students to have more practical opportunities, increase students’ confidence, and thus reduce students’ perceived pressure and learning burnout level.

## Data Availability

The raw data supporting the conclusions of this article will be made available by the authors, without undue reservation.
